# Effects of low to moderate levels of deoxynivalenol on feed and water intake, weight gain, and slaughtering traits of broiler chickens

**DOI:** 10.1007/s12550-017-0284-z

**Published:** 2017-07-07

**Authors:** A. Lucke, B. Doupovec, P. Paulsen, Q. Zebeli, J. Böhm

**Affiliations:** 1 Institute of Animal Nutrition and Functional Plant Compounds, Department for Farm Animals and Veterinary Public Health, University of Veterinary Medicine Vienna, Veterinärplatz 1, 1210 Vienna, Austria; 2 BIOMIN Research Center, Technopark 1, 3430 Tulln, Austria; 3 Institute of Meat Hygiene, Meat Technology and Food Science, Department for Farm Animal and Veterinary Public Health, University of Veterinary Medicine Vienna, Veterinärplatz 1, 1210 Vienna, Austria

**Keywords:** Broiler, Deoxynivalenol (DON), Performance, Water intake

## Abstract

The aim of the study was to evaluate the effects of low to moderate oral exposure to the *Fusarium* toxin deoxynivalenol (DON; derived from culture material) on performance, water intake, and carcass parameters of broilers during early and late developmental phases. A total of 160 Ross 308 broilers were randomly allocated to four different feeding groups (*n* = 40/group) including 0 (control), 2.5, 5, and 10 mg DON/kg wheat-soybean meal-based feed. Three consecutive replicates of the experiment were performed. Half of the broilers were slaughtered in week 3 of the trial whereas the other half were slaughtered in week 5. Dry matter intake (DMI) and water intake (WI) were recorded on a daily basis and the body weight (BW) and BW gain (BWG) were determined weekly. The following carcass traits were recorded and calculated in absolute and relative data: dressed carcass weight, breast muscle weight, leg weight, and liver weight. Data showed that BW (*P* < 0.001), BWG (*P =* 0.005), and DMI (*P* < 0.001) were reduced by DON-feeding during the entire feeding period. The ratio of DMI to body weight gain (DMI/BWG) was not affected by the treatment. However, the ratio of water to DMI (WI/DMI) increased in DON-treated birds (*P =* 0.021). Contrast analysis showed that DON tendentially reduced slaughter weight (*P* = 0.082) and decreased leg yield (*P* = 0.037) in DON-fed chickens in week 5 of the experiment. Liver organ weight decreased in the 3-week-old DON-fed broilers compared to that in the control-fed birds (*P* = 0.037). In conclusion, the study suggests that DMI and BW were negatively affected under the experimental conditions at DON levels lower than the current guidance value in the European Union of 5 mg/kg feed. The study also indicates that broilers fed on low to moderate level DON-contaminated diets showed increased WI/DMI ratio which might have negative influence on wet litter syndrome.

## Introduction

Deoxynivalenol (DON) is one of the most widespread trichothecene toxins in grains, such as wheat, rye, barley, maize, and oats, as well as their by-products and other commodities used in feed production (Escriva et al. 2015). Indeed, a recent survey made from Streit et al. (2013), evaluating feed samples from 2004 to 2011 around the world, with a special focus on Europe and Asia, determined that DON was the most prevalent mycotoxin, with 55% of the samples testing positive. Contamination of feeds with low and moderate doses of DON is therefore a big health concern in non-ruminant livestock production.

Harmful effects of DON contamination on gastrointestinal health in poultry are well known (Awad et al. 2013; Ghareeb et al. 2015). The effects of DON on broiler performance were examined in several studies too, and the results obtained were inconsistent (Awad et al. 2004, 2006a; Dänicke et al. 2003; Kubena et al. 1988, 1989). While Awad et al. (2006a) found no adverse effects of feeding contaminated feed with 10 mg DON/kg feed on bodyweight and feed consumption on group-housed Ross E032-broilers, the study by Dänicke et al. (2003) revealed linearly decreased feed intake and weight gain with increasing dietary proportions of DON-contaminated wheat with concentrations of 3.5 to 14 mg DON/kg feed in the diet of male Lohmann broilers reared for 5 weeks. Overall, meta-analytical data suggest a reduction of weight gain of 1.2% per milligram deoxynivalenol per kilogram diet (Andretta et al. 2011). Currently, the guidance level for DON in the European Union in complete feed for poultry is set at 5 mg DON/kg feed (EC 2006). Eriksen and Pettersson (2004) summarized that a level of 2.5 mg DON/kg feed can be considered as safe in chicken feed. However, the toxigenic effects of DON in poultry depend not only on the dose but also on the length of exposure to DON and also on other factors whereby the interactions with other dietary components affecting intestinal digestion and health seem to play a role (Ghareeb et al. 2015).

In addition, differences in metabolic and growth rates of animals have been suggested as one reason of the inconsistency of the results obtained with studies which used different levels of DON but also birds with different genetic growth potential and feed efficiency traits. For example, Yunus et al. (2012) suggested that earlier research has been conducted with different broiler strains and modern broilers have a higher feed efficiency compared to broilers of previous decades (Dozier et al. 2008) which could have an impact on the level at which mycotoxins affect the performance (Yunus et al. 2011, 2012). Considering the industrial aim to produce high-performing broilers, it is important to reevaluate threshold levels for DON in chicken feed which are reflected in the currently applicable guidance values (EC 2006). Therefore, studies examining the effects of low to moderate levels of DON on performance parameters of broilers are indicated.

Wet litter is a serious problem in broiler chickens and can be associated with food pad dermatitis which results in welfare issues and economic losses for the broiler industry (Dunlop et al. 2016). Increased water intake per unit of feed intake can be a cause for increased moisture content in litter. Mycotoxins like ochratoxin A, citrinin, and oosporin have nephrotoxic effects and are linked to wet litter problems (Collett 2012; Hoer 2008). Awad et al. (2014) observed decreased absolute and relative kidney weights in broilers after 5 weeks of DON-feeding as a sign of possible kidney damage. To our knowledge, the effects of DON on water intake in broilers have not been studied yet. The aim of the study was to elucidate the effects of practically relevant levels of DON from 2.5 to 10 mg/kg feed on water and dry matter intake and their relation to each other as well as to body weight performance and carcass traits.

## Materials and methods

### Animals and experimental design

In total, 160 1-day-old broiler chicks (ROSS 308) were purchased from a commercial hatchery (Brüterei Schlierbach GmbH, Steinbachbrücke 35, 4643 Pettenbach, Austria) and were randomly allocated to one of the four feeding groups containing or not experimentally contaminated DON feed as follows: (1) control diet without DON, (2) control diet experimentally contaminated with 2.5 mg DON (Romer Labs, Technopark 1, 3430 Tulln, Austria)/kg feed (2.5 mg/kg–DON group), (3) control diet contaminated with 5 mg DON/kg feed (5 mg/kg–DON group), and (4) control diet contaminated with 10 mg DON/kg feed (10 mg/kg–DON group).

The trial was performed in three experimental runs with 32, 64, and 64 broilers each. The animals were kept in 16 flat deck cages with 0.36 m^2^ each. In every cage, four chickens were kept together for the first 3 weeks and two chickens were kept for the last 2 weeks of each experimental run. In the run with 32 broilers, the number of birds per cage was two for the first 3 weeks and one for the remaining time.

Animals were provided with 23 h of light for the first 6 days, followed by 2 days with a 22-h light period and were kept at 20 h of light for the rest of the trial. Temperature management was according to the Ross Aviagen Management Handbook (Aviagen 2014a). Birds were spray-vaccinated (Poulvac IB-Primer, Zoetis, 10785 Germany) by the supplier and have been individually vaccinated with a live vaccine against infectious bronchitis (Nobilis IB 4-91, MSD Animal Health, Intervet International, 5831 Boxmeer, Netherlands), Newcastle disease (AviPro ND La Sota Lohmann Animal Health GmbH, 27472 Cuxhaven, Germany), and Gumboro (AviPro Gumboro Vac Cu-1M, Lohmann Animal Health GmbH, 27472 Cuxhaven, Germany).

In each treatment group, half of the animals were slaughtered in week 3 (day 20 to 23) while the other half of the birds were slaughtered in week 5 (day 34 to 37). The animal trial was approved by the institutional ethics and animal welfare committee and the national authority according to §§ 26 of Animal Experiments Act, Tierversuchsgesetz 2012–TVG 2012 (file number: BMWFW–68.205/0062–WF/V/3b/2015).

### Diets and feeding

The control diet was a wheat/soy-based diet supplemented with a commercially available mineral-vitamin supplement (Biomin BR 15% CAN, Biomin GmbH, Getzersdorf, Austria). The dietary ingredients and chemical composition are shown in Table 1. The experimentally contaminated diets were mixed in three steps fresh for each experimental run. First, purified DON culture material was mixed with inulin at a rate of 0.03 to 0.20% in order to reach the required DON dosage in the premix. Inulin was chosen as carrier to ensure homogenous distribution of DON in the diet and was maintained constant across all treatments (Masching et al. 2016). The following premix was subsequently mixed by including the total of 250 g basal premix and mixing it with 750 g of the control feed to deliver the premix for contaminated feed. The latter premix was then mixed with the final amount of feed for each experimental group per run, delivering an average contamination of 0.16 mg DON/kg feed for control, 1.83, 5.00, and 9.45 mg DON/kg feed for the 2.5, 5, and 10 mg/kg groups, respectively.

**Table 1:** Ingredients and nutrient composition of the basal diet

Ingredients (kg per 100 kg feed)	
Wheat (11% crude protein)	57.59
Soy bean meal	25.44
Supplement^a, b^	13.00
Rapeseed oil	2.97
Megafat	1
Analytical composition (g per kg fresh matter)	
Dry matter	889
Crude protein	207
Crude fat	78
Crude fiber	30
Crude ash	73
Starch	365
Sugar	52
Calculated metabolizable energy (MJ/kg)	12.66

^a^Composition of the supplement: soybean, toasted 46.2%; calcium carbonate 13%; monocalcium phosphate 12.6% hardened palm kernel oil 12.5%; pumpkin seed cake 4.8%; sodium bicarbonate 2.3%; sodium chloride 1.5%; magnesium phosphate 0.9%

^b^Per kilogram product: dry matter, 94%; calcium 7.2%; phosphorus 3.3%; sodium 1.2%; iron 850 mg; copper 160 mg; zinc 500 mg; manganese 500 mg; iodine 16.6 mg; selenium 3 mg; vitamin A 90000 IE; vitamin D3 33,300 IE; vitamin E 650 mg; vitamin K3 25 mg; vitamin B1 25 mg; vitamin B2 60 mg; vitamin B6 50 mg; vitamin B12 300 μg; pantothenic acid 115 mg; nicotinic acid 610 mg; vitamin C 600 mg; folic acid 16 mg; biotin 1800 μg; choline chloride 4700 mg; lysine 2.9%; methionine 1.9%; threonine 1.5%; butylated hydroxytoluene (E321) 1000 mg

Birds had free access to drinking water and feed during the whole trial. The feed was offered in plastic feeders (1.5 kg; article number FA69364; FAIE Handelsgesellschaft mbH, 4844 Regau, Austria) in mashed form. Chicken plastic drinkers (1.5 l, article number 5295, Siepmann GmbH, 58313 Herdecke, Germany) were cleaned daily and refilled with tap water. To avoid contamination of the drinking water with feed, drinkers were placed apart from the feeders. Feed and water intakes were measured daily at 14:00 by measuring the weight difference of the feeder and drinker compared to the previous measurement, respectively. The daily feed intake per bird (FI_bird_) was calculated by dividing the feed intake per cage by the number of animals present in the cage: FI_bird_ = FI_cage_/number of animals per cage. The feed intake was then transformed to dry matter intake (DMI) by multiplying the daily feed intake by the dry matter (DM) content of the feed (%): DMI_bird_ = FI_bird_ × DM. The daily water intake per bird (WI_bird_) was calculated by dividing the daily water intake per cage by the number of animals present in the same cage: WI_bird_ = WI_cage_/number of animals per cage. The body weight (BW) was recorded weekly as a single animal measurement on days 1, 7, 14, 21, 28, and 35. The ratio of dry matter intake to body weight gain (DMI/BWG) was calculated by addition of the consumed feed (as dry matter) between two time points of body weight measurements divided by the body weight gain (BWG) in one experimental week: DMI/BWG_weekY_ = DMI_birdX in weekY_/BWG_birdX in weekY_.

### Feed samples and analyses

The basal diet was analyzed by proximate nutrient analysis including DM, crude protein, crude fat, crude fiber, crude ash, starch, and sugar by Futtermittellabor Rosenau, Gewerbepark Haag 3, 3250 Wieselburg-Land, Austria (Table 1). Feed samples taken in week 3 of every experimental run were used to measure DM which was used to calculate the DMI. Dry matter was measured by drying the feed in a hot air oven overnight at 103 °C. Analyses of DON in feed samples were carried out with HPLC-MS/MS by Romer Labs Diagnostic GmbH (3430 Tulln, Austria).

### Slaughtering procedure

The birds were injected 50–100 mg/kg BW thiopental natrium (Thiopental medicamentum, medicamentum pharma GmbH, 8643 Allerheiligen im Mürztal, Austria) intravenously after fasting for 0.5 to 1.5 h and were subsequently euthanized by exsanguination. After degutting, removal of the liver, kidney, trachea, esophagus, crop, and head, the carcass was skinned, the legs were cut in the tarsal joint, and the neck was removed. In this condition, the weight of the dressed carcass was determined. The legs (i.e., thigh plus drumstick, including bones) and breast muscles (deep and superficial part of the pectoralis muscle excluding meat in the furcula region) were removed and weighed. Liver weight was determined. Cutting was done within 3 h post mortem.

### Statistical analyses

Statistical analyses were performed with PROC MIXED of SAS version 9.4 using different models. The ANOVA model of DMI, WI, and WI/DMI ratio data accounted for the fixed effects of run, week, and DON treatment as well as the interaction of DON treatment × week. Run was considered as random effect. Measurements taken on the same experimental unit but on different days were considered as repeated measures in the ANOVA using an autoregressive covariance structure, according to Bayesian Information Criteria. Contrasts of DON effects were tested using the contrast statement of SAS. Tukey-Kramer corrections were applied to compare multiple means.

For the data of BW, the initial BW at day 1 has been considered as a covariate in the model. If this was not significant, the covariate was removed by the model. The fixed effects in the ANOVA for BW, BWG, and DMI/BWG ratio were week, DON treatment, and the interaction of DON treatment × week. Measurements taken on the same animal but on different weeks were considered as repeated measures with the covariance structure being compound symmetry, according to Bayesian Information Criteria. Run and the cage were considered as random effects. Contrasts of DON levels in the diet were tested using the contrast statement of the SAS. Tukey-Kramer corrections were applied to compare multiple means.

Slaughtering traits were analyzed separately for week 3 and week 5. The fixed effect in the ANOVA was DON treatment and the animal within run was considered as a random effect with the covariance structure being variance components, according to Bayesian Information Criteria. Orthogonal, linear, and quadratic contrasts were tested using the contrast statement of SAS.

Degrees of freedom were computed with the Kenward-Roger-method, and data are presented as least square means (LS means) ± standard error of the mean (SEM). Effects of run and the interaction of DON treatment and run were tested for each variable separately. Results were considered to be statistically significant if *P* < 0.05, and a tendency was considered at 0.05 < *P* < 0.10.

## Results

### Body weight, body weight gain, dry matter intake, and ratio of dry matter intake to body weight gain

The start weight of broiler chicks did not differ significantly among the groups at day 1 (33.8, 34.7, 35.0, and 32.8 g in the control, 2.5 mg/kg, 5 mg/kg, and 10 mg/kg groups, respectively). The BW over the course of the experiment is shown in Table 2. Data showed that the BW was decreased in DON-fed animals with a significantly different contrast comparing the control-fed animals to all DON groups (*P* < 0.001, Table 2). Overall, the control group weighed 729.8 g whereas the 2.5 and 5 mg/kg DON groups had a decreased BW of 692.5 and 686.6 g, respectively (*P* = 0.010 and *P* = 0.002). The weekly BWG was also decreased by DON treatment (*P* = 0.005). There were no significant differences in BWG in the single weeks. However, the overall weekly BWG in the 5 mg/kg DON group was decreased compared to the control group (*P* = 0.003).

**Table 2:** Body weight, body weight gain per week, and ratio of dry matter intake to body weight gain (Lucke et al. 2016)

	Body weight (g)^c^	Body weight gain (g/week)	Ratio dry matter intake to body weight gain (g/g)^d^
Week 1			
Control	131.3 ± 21.4	94.7 ± 13.4	1.29 ± 0.15
2.5 mg/kg	123.1 ± 21.6	86.0 ± 13.5	1.36 ± 0.15
5 mg/kg	128.9 ± 21.5	91.9 ± 13.5	1.31 ± 0.15
10 mg/kg	124.5 ± 21.6	88.3 ± 13.5	1.38 ± 0.15
Week 2			
Control	388.1 ± 21.4	254.0 ± 13.4	1.46 ± 0.15
2.5 mg/kg	369.4 ± 21.6	244.0 ± 13.5	1.45 ± 0.15
5 mg/kg	371.2 ± 21.5	240.2 ± 13.5	1.49 ± 0.15
10 mg/kg	383.7 ± 21.6	255.7 ± 13.5	1.40 ± 0.15
Week 3			
Control	776.4 ± 21.7	386.6 ± 13.7	1.43 ± 0.15
2.5 mg/kg	741.9 ± 21.9	372.1 ± 13.8	1.43 ± 0.15
5 mg/kg	755.4 ± 21.7	379.1 ± 13.7	1.44 ± 0.15
10 mg/kg	763.5 ± 21.9	375.5 ± 13.8	1.42 ± 0.15
Week 4			
Control	1256.0 ± 24.1	468.5 ± 15.5	1.68 ± 0.16
2.5 mg/kg	1183.0 ± 24.7	437.7 ± 15.8	1.62 ± 0.16
5 mg/kg	1181.1 ± 24.1	422.8 ± 15.4	1.67 ± 0.16
10 mg/kg	1220.1 ± 24.2	455.8 ± 15.5	1.58 ± 0.16
Week 5			
Control	1793.3^a^ ± 25.3	528.6 ± 16.3	1.77 ± 0.16
2.5 mg/kg	1703.2^ab^ ± 26.2	497.7 ± 16.9	1.71 ± 0.16
5 mg/kg	1647.9^b^ ± 25.3	470.1 ± 16.3	1.73 ± 0.16
10 mg/kg	1711.6^ab^ ± 25.4	482.4 ± 16.4	1.75 ± 0.16
Overall			
Control	729.8^a^ ± 19.5	346.5^a^ ± 12.1	1.53 ± 0.15
2.5 mg/kg	692.5^b^ ± 19.6	327.5^ab^ ± 12.1	1.51 ± 0.15
5 mg/kg	686.6^b^ ± 19.5	320.8^b^ ± 12.1	1.53 ± 0.15
10 mg/kg	706.0^ab^ ± 19.6	331.5^ab^ ± 12.2	1.51 ± 0.15
*P* values^e^			
Week	<0.001	<0.001	<0.001
DON treatment	0.001	0.005	0.965
DON treatment × week	<0.001	0.152	0.848
Contrasts			
Control vs. DON	<0.001	0.001	0.804
Contrast linear	0.037	0.028	0.790
Contrast quadratic	0.001	0.005	0.892

Data are presented as least square means (LSM) ± standard error of the mean (SEM)

^a, b^Values with no common superscripts differ significantly within week and columns (*P* < 0.05)

^c^The body weight was measured individually. The weight gain between the measurements was calculated

^d^The ratio of dry matter intake to body weight gain was calculated by dividing the sum of consumed dry matter per bird between two measurements of body weight by the body weight gain

^e^The *P* values for effects of run and run × treatment are 0.420 and 0.998 for body weight, 0.002 and 0.737 for body weight gain, and <0.001 and 0.889 for the ratio of dry matter intake and body weight gain, respectively

Comparing the least square means of the BW of the feeding groups within each experimental week, the 5 mg/kg DON group had decreased BW in week 5 compared to that of the control group (1647.9 and 1793.3 g, respectively; *P* < 0.001). No significant differences between the treatment groups were found within weeks 1 to 4 of the experiment.

Table 3 summarizes the data for DMI, WI, and WI/DMI. Overall effects showed that daily DMI was decreased in all DON-contaminated groups compared to that in the control group (*P* < 0.001). Comparing the DMI per day of the different groups per day within each week, a decreased DMI was observed in week 5 for the 5 mg/kg and the 10 mg/kg DON/kg feed groups compared to that in the control group (*P* = 0.002 and *P* = 0.002, respectively).

**Table 3:** Daily dry matter intake and water intake and ratio of water to dry matter intake per bird (Lucke et al. 2016)

	Dry matter intake per bird (g/day)^c^	Water intake per bird (g/day)^d^	Ratio water intake to dry matter intake^e^
Week 1			
Control	23.13 ± 2.66	52.20 ± 6.18	2.53 ± 0.09
2.5 mg/kg	22.95 ± 2.66	45.91 ± 6.18	2.42 ± 0.09
5 mg/kg	23.02 ± 2.66	49.44 ± 6.18	2.61 ± 0.09
10 mg/kg	23.21 ± 2.66	48.07 ± 6.21	2.41 ± 0.09
Week 2			
Control	59.11 ± 2.65	102.44 ± 5.00	1.92 ± 0.07
2.5 mg/kg	53.34 ± 2.65	99.75 ± 4.99	1.98 ± 0.07
5 mg/kg	54.62 ± 2.65	99.16 ± 4.99	1.99 ± 0.07
10 mg/kg	55.09 ± 2.65	100.38 ± 4.99	1.93 ± 0.07
Week 3			
Control	78.79 ± 2.65	166.62 ± 5.00	2.16 ± 0.07
2.5 mg/kg	74.53 ± 2.65	158.98 ± 4.98	2.19 ± 0.07
5 mg/kg	76.17 ± 2.65	162.75 ± 5.02	2.22 ± 0.07
10 mg/kg	76.49 ± 2.65	164.53 ± 5.00	2.20 ± 0.07
Week 4			
Control	111.60 ± 2.65	219.87 ± 4.98	2.05 ± 0.07
2.5 mg/kg	101.30 ± 2.65	221.75 ± 4.98	2.23 ± 0.07
5 mg/kg	100.80 ± 2.65	219.18 ± 5.00	2.34 ± 0.07
10 mg/kg	102.64 ± 2.65	221.48 ± 4.98	2.18 ± 0.07
Week 5			
Control	131.31^a^ ± 2.69	263.32 ± 5.05	2.12 ± 0.07
2.5 mg/kg	120.81^ab^ ± 2.69	270.70 ± 5.07	2.31 ± 0.08
5 mg/kg	114.48^b^ ± 2.69	264.44 ± 5.10	2.42 ± 0.08
10 mg/kg	114.54^b^ ± 2.69	255.37 ± 5.05	2.33 ± 0.07
Overall			
Control	80.79^a^ ± 1.22	160.89 ± 2.42	2.16^a^ ± 0.04
2.5 mg/kg	74.59^b^ ± 1.22	159.42 ± 2.42	2.22^ab^ ± 0.04
5 mg/kg	73.82^b^ ± 1.22	158.99 ± 2.43	2.31^b^ ± 0.04
10 mg/kg	74.39^b^ ± 1.22	157.96 ± 2.42	2.21^ab^ ± 0.04
P values^f^			
Week	<0.001	<0.001	<0.001
DON treatment	<0.001	0.859	0.021
DON treatment × week	0.109	0.909	0.703
Contrasts			
Control vs. DON	<0.001	0.452	0.025
Contrast linear	< 0.001	0.394	0.113
Contrast quadratic	0.006	0.927	0.018

Data are presented as least square means (LSM) ± standard error of the mean (SEM)

^a, b^Values with no common superscripts differ significantly within week and columns (*P* < 0.05)

^c^Feed consumption was recorded daily per cage, divided by the number of animals in the cage and converted in dry matter intake

^d^Water intake was measured daily per cage and was divided by the number of animals in the cage

^e^The ratio of water to dry matter intake was determined by dividing the daily dry matter intake (g/bird) by the daily water intake (g/bird)

^f^The *P* values for effects of run and run × treatment are 0.468 and 1.000 for dry matter intake, 0.617 and 0.999 for water intake, and <0.001 and 0.045 for the ratio of water intake to dry matter intake, respectively

The ratio of DMI/BWG was not altered during the whole period of the experiment and within each of the experimental weeks (Table 2).

### Water intake and ratio of water to dry matter intake

The overall absolute WI per day was not affected by the DON treatment (*P* = 0.859). Within the same experimental weeks, WI was not influenced significantly (Table 3). However, DON increased the ratio of WI/DMI (*P* = 0.021). Overall, the 5 mg/kg DON group showed an increased ratio of WI/DMI compared to that of the control group (*P* = 0.011) and a significant quadratic effect of DON on WI/DMI (*P* = 0.018) was observed.

### Carcass parameters

Absolute and relative carcass traits (as percentage of the body weight) are shown in Tables 4 and 5, respectively. The slaughter weight, defined as the live weight of the birds measured directly before slaughter, was tendentially decreased in DON-treated birds in week 5 compared to that of control birds (*P* = 0.082). Both absolute and relative weights of the dressed carcass were not affected by the treatment at any time point. Absolute and relative breast muscle weights were not affected by the treatment. The absolute weight of broiler legs was decreased by DON treatments in week 5 of the experiment in comparison to that of control treatment (*P* = 0.037, Table 4). In week 5, broiler legs in the 5 mg/kg DON group were lighter than those of the control group (307 and 336 g, respectively; *P* = 0.031). Relative weights of broiler legs were not affected by the treatment.

**Table 4:** Carcass traits as absolute weights (g)

	Slaughter weight^c^	Dressed carcass^d^	Breast muscle^e^	Leg weight^f^	Liver weight
3 weeks					
Control	795 ± 27	434 ± 16	128 ± 5	132 ± 5	20.9 ± 0.8
2.5 mg/kg DON	770 ± 27	422 ± 16	119 ± 5	128 ± 5	18.9 ± 0.8
5 mg/kg DON	773 ± 28	429 ± 16	122 ± 6	130 ± 5	19.0 ± 0.8
10 mg/kg DON	783 ± 28	433 ± 16	123 ± 6	133 ± 5	18.9 ± 0.8
*P* values^g^					
DON treatment	0.916	0.951	0.670	0.893	0.217
Contrasts					
Control vs. DON	0.536	0.767	0.272	0.806	0.037
Contrast linear	0.794	0.932	0.617	0.783	0.114
Contrast quadratic	0.531	0.627	0.337	0.487	0.245
5 weeks					
Control	1812 ± 53	1110 ± 35	345 ± 14	336^a^ ± 9	34.3 ± 1.6
2.5 mg/kg DON	1708 ± 53	1064 ± 35	335 ± 14	317^ab^ ± 9	33.2 ± 1.6
5 mg/kg DON	1691 ± 53	1038 ± 35	340 ± 14	307^b^ ± 9	31.5 ± 1.6
10 mg/kg DON	1712 ± 53	1051 ± 35	342 ± 14	315^ab^ ± 9	31.0 ± 1.6
*P* values^h^					
DON treatment	0.368	0.505	0.966	0.167	0.439
Contrasts					
Control vs. DON	0.082	0.152	0.725	0.037	0.196
Contrast linear	0.186	0.201	0.968	0.089	0.110
Contrast quadratic	0.244	0.411	0.663	0.145	0.833

Data are presented as least square means (LSM) ± standard error of the mean (SEM)

^a, b^Values with no common superscripts differ significantly within columns (*P* < 0.05)

^c^The slaughter weight was determined as the weight of the living bird before exsanguinations

^d^The dressed carcass weight was eviscerated and skinned and the neck and legs distal from the tibiotarsal joint were removed

^e^Breast muscle weight includes the deep and superficial part of the pectoralis muscle excluding meat in the furcula region

^f^Leg weight includes left and right thighs with drumstick and bones

^g^The *P* values in week 3 for effects of run and run × treatment are 0.099 and 0.978 for slaughter weight, 0.566 and 0.994 for dressed carcass, 0.835 and 0.892 for breast muscle, 0.07 and 0.999 for leg weight, and 0.054 and 0.611 for liver weight, respectively

^h^The *P* values for effects of run and run × treatment are <0.001 and 0.330 for slaughter weight, <0.001 and 0.392 for dressed carcass, 0.004 and 0.999 for breast muscle, 0.001 and 0.150 for leg weight, and <0.001 and 0.490 for liver weight, respectively

**Table 5:** Carcass traits as relative weights (% of slaughter weight)

	Dressed carcass^c^	Breast muscle^d^	Leg weight^e^	Liver weight
3 weeks				
Control	54.6 ± 0.7	16.1 ± 0.4	16.6 ± 0.2	2.63^a^ ± 0.07
2.5 mg/kg DON	54.7 ± 0.7	15.4 ± 0.4	16.7 ± 0.2	2.45^ab^ ± 0.07
5 mg/kg DON	55.4 ± 0.8	15.6 ± 0.4	16.8 ± 0.2	2.44^ab^ ± 0.08
10 mg/kg DON	55.4 ± 0.8	15.7 ± 0.4	17.0 ± 0.2	2.40^b^ ± 0.08
*P* values^f^				
DON treatment	0.796	0.658	0.420	0.143
Contrasts				
Control vs. DON	0.500	0.264	0.336	0.024
Contrast linear	0.352	0.619	0.110	0.040
Contrast quadratic	0.942	0.307	0.612	0.390
5 weeks				
Control	61.2 ± 0.4	19.1 ± 0.5	18.6 ± 0.2	1.86 ± 0.06
2.5 mg/kg DON	62.1 ± 0.4	19.5 ± 0.5	18.6 ± 0.2	1.91 ± 0.06
5 mg/kg DON	61.3 ± 0.4	20.1 ± 0.5	18.2 ± 0.2	1.84 ± 0.06
10 mg/kg DON	61.4 ± 0.4	19.9 ± 0.5	18.5 ± 0.2	1.82 ± 0.06
*P* values^g^				
DON treatment	0.456	0.550	0.533	0.698
Contrasts				
Control vs. DON	0.477	0.229	0.493	0.975
Contrast linear	0.825	0.207	0.464	0.482
Contrast quadratic	0.342	0.565	0.491	0.481

All weights are presented as percentage of the slaughter weight. Data are presented as least square means (LSM) ± standard error of the mean (SEM)

^a, b^Values with no common superscripts differ significantly within columns (*P* < 0.05)

^c^The dressed carcass weight was eviscerated and skinned and the neck and legs distal from the tibiotarsal joint were removed

^d^Breast muscle weight includes the deep and superficial part of the pectoralis muscle excluding meat in the furcula region

^e^Leg weight includes left and right thigh with drumstick and bones

^f^The *P* values in week 3 for effects of run and run × treatment are <0.001 and 0.850 for dressed carcass, 0.004 and 0.589 for breast muscle, 0.541 and 0.134 for leg weight, and 0.046 and 0.416 for liver weight, respectively

^g^The *P* values in week 5 for effects of run and run × treatment are 0.005 and 0.037 for dressed carcass, 0.130 and 0.710 for breast muscle, 0.007 and 0.658 for leg weight, and 0.232 and 0.356 for liver weight, respectively

### Liver weight

Absolute and relative liver weights were decreased comparing DON-fed animals with control-fed animals in week 3 (*P* = 0.037 and *P* = 0.024, respectively). The relative liver weight in week 3 decreased in a linear fashion (*P* = 0.040). Furthermore, the 10 mg/kg group had a decreased relative liver weight compared to the control group (2.40 and 2.63%, respectively; *P* = 0.034).

## Discussion

The aim of this study was to evaluate the effects of low to moderate DON concentrations in broiler feed on performance parameters and WI and determine their role on carcass traits. In this study, a decreased BW and DMI were detected for DON-treated birds compared to those of the control-fed chickens. Overall, the least square means of the BW were decreased in the 2.5 and 5 mg/kg DON-treated animals in our trial even though DON in concentrations of up to 8 mg/kg (Pestka 2007) or 16 mg/kg (Dersjant-Li et al. 2003) were not reported to affect productivity in poultry. Interestingly, until week 4 of the experiment, BW did not differ significantly among the feeding groups. In week 4, DON-fed birds showed a numerically decreased BW and in week 5, the 5 g/kg DON-fed birds had a significantly lower BW compared to that of the control group. Furthermore, in our study, the effects of DON on the BW were more pronounced in the 2.5 and 5 mg/kg DON-fed broilers. This is in accordance with the overall decreased BWG in the 5 mg/kg DON group (*P* = 0.03). This result is of practical relevance. The current guidance value for DON in complete feedingstuffs for poultry is set at 5 mg/kg feed (EC 2006). In this study, we showed that DON concentrations lower than the recommended guidance value can have a negative impact on broiler performance.

In this study, the DMI showed a pattern corresponding to the BW development. In line with the BW development, the daily DMI was numerically decreased in week 4 of the experiment. In the last experimental week, the daily DMI was reduced in all DON-fed birds, reaching a significant level in the 5 and 10 mg/kg DON-fed birds. As the ratio of DMI/BWG was not significantly influenced by the treatment at any time point of the experiment, it can be concluded that the lowered DMI resulted directly in reduced BW of the chickens. Trichothecenes were reported to reduce food intake by the induction of gastrointestinal malaise and by increasing satiety (Lebrun et al. 2015).

Ghareeb et al. (2012) reported decreased feed intake in broilers starting at week 3 of the experiment using a corn-soy-based diet which was supplemented with 10 mg DON/kg feed, after changing from starter to the low-protein grower diet. Awad et al. (2006b) observed a significantly decreased feed intake in the last week of a 3-week experiment in broilers fed with 5 mg DON/kg feed. Their study used naturally contaminated wheat which was incorporated at a proportion of 50% into the diet. Similar to our study, Awad et al. (2006b) used one diet over the experimental period. In the study of Swamy et al. (2002), it was shown that *Fusarium* mycotoxin-contaminated diets caused a significant response of feed intake and BWG in a quadratic fashion in the finisher period (Swamy et al. 2002). In the mentioned experiment, DON concentrations of up to 11 mg/kg were used, while fusaric acid and zearalenone ranged between 14 to 26 mg/kg and <0.1 to 1.2 mg/kg, respectively (Swamy et al. 2002).

Generally, the information on feed intake and BW data during DON exposure is inconsistent. Besides those studies which found a decreased feed intake at low to moderate doses of DON towards the end of the experiment (Awad et al. 2006b; Ghareeb et al. 2012), other authors did not find a statistically significant effect of DON treatment on feed intake and BW (Antonissen et al. 2015; Awad et al. 2004). Ghareeb et al. (2014) found numerical decreases in feed intake and BW which did not reach statistical significance in a 10 mg/kg DON-contaminated maize-soy-based diet.

On the other hand, Yunus et al. (2012) found that increasing DON decreased the weekly BWG in a linear fashion in the first 3 weeks of exposure while feeding either a control diet, a low DON diet (1.68 mg DON/kg feed), or a high DON diet (12.209 mg DON/kg feed) from day 7 to day 35 to male broiler chicks. In that study, the weekly weight gain was lower in weeks 1 to 3 in the high DON group whereas the low DON group only showed decreased weight gain during week 3 of the exposure (Yunus et al. 2012). No significant differences for the BWG were found after week 3, which was explained by the fact of a smaller number of data points available at later stages during the experiment (Yunus et al. 2012). The feed consumption in the study of Yunus et al. (2012) was affected for both low and high DON groups in week 3 of the experiment but overall the feed consumption up to week 3 and week 5 was decreased compared to that of the basal diet only for the high DON group.

Awad et al. (2011) found a significantly (quadratic) decreased feed intake in week 1 for chicks fed a DON-contaminated diet compared to that of chickens fed a control diet in an experiment with three dietary treatments: control diet, 1 mg DON/kg, and 5 mg DON/kg for 5 weeks. Following this, in the second experimental week, a significant (quadratic) decreased BW was found. However, the mean BW and feed intake over the course of the experiment were not affected by the DON treatment (Awad et al. 2011).

It can be concluded that changes in feed intake and BWG depending on DON contamination are inconsistent between different studies. Therefore, differences in diets and further stressors over the course of the experiment to the broilers have to be taken into account. In our experiment, broilers were fed with one diet throughout the whole experimental period, without changing from a starter to a grower and finisher diet. Consequently, the broilers were fed on a relatively low protein level in their first week of age (20.7% in the fresh matter) (Kamphues et al. 2014). Furthermore, physical form of the presented feed should also be considered when comparing broiler performances in different studies. In our study, broilers were fed a mashed diet. Many studies showed that pelleting can improve broiler performance (Amerah et al. 2007; Jahan et al. 2006; Serrano et al. 2013). However, as all treatment groups received the same physical form of diet (mash) in our study, it is not expected that the effects of DON are altered due to feeding mash instead of pellets. These factors might also explain the relatively low BW compared to the expectations for ROSS 308 broilers from 2144 g body weight for 35-day-old broilers (Aviagen 2014b). Furthermore, the broilers were subjected to three vaccinations associated with single-bird treatments in early life which could have resulted in psychological stress on the one hand and immunological stress on the other hand. For commercial poultry production, decreases in performance even at low contamination levels, should be a concern, as additional stressors to the birds as vaccinations, or mass production cannot be excluded and might lead to more pronounced effects and consequently economic losses than DON contamination alone.

In the present study, DON-treated broilers showed a significantly increased WI/DMI. During weeks 4 and 5 of the experiment, the DON groups showed numerically increased WI/DMI. Overall, WI/DMI was increased in the 5 mg/kg DON group while the 2.5 and 10 mg/kg DON groups showed a numerical increase. To our knowledge, the relation between water intake and DON-contaminated feed has not yet been evaluated in chickens. It has been previously shown that a naturally *Fusarium* mycotoxin-contaminated diet can lead to an increase in water consumption in rabbits (Hewitt et al. 2012). Wet litter poses a major problem in meat-type chickens and one of the many key contributing factors is increased water consumption (Dunlop et al. 2016). It is known that mycotoxins like ochratoxin A, citrinin, and oosporin have nephrotoxic potential (Collett 2012; Hoer 2008). For further investigations concerning changes in water consumption behavior in broilers, the mode of contamination (comparing naturally contaminated feed with artificially contaminated feed) might also be of interest. In this study, we used an artificially DON-contaminated diet. In contrast, in naturally contaminated feed, co-occurrence of several mycotoxins is common as most fungi have the ability to produce several mycotoxins and as commodities may be infected by several fungi (Streit et al. 2012). This could potentially lead to synergistic or additive effects (Streit et al. 2012).

In chickens, it has been previously shown, that DON reduces both absolute and relative organ weight of the kidney (Awad et al. 2014). The authors of the previously mentioned study therefore suggest that these findings could indicate that DON damages the kidney cells. In contrast, another study reported no significant alteration of the relative kidney weight in DON-treated animals but showed a numerical increase (Frankic et al. 2006). Feeding different levels of deoxynivalenol, fusaric acid, and zearalenone to broilers were shown to linearly increase serum uric acid concentration at day 56 (Swamy et al. 2002). Further research elucidating the interplay between the elevated WI/DMI and possible kidney damage is warranted. This is especially the case, as other mycotoxins like ochratoxin A, citrinin, and oorsporin have to be considered in the case of wet litter problems (Collett 2012). Therefore, the elevated WI/DMI might be of practical relevance for the broiler industry both in terms of animal welfare and economic reasons.

In this study, absolute and relative weights of the dressed carcass and breast muscles were not affected by the dietary treatment. The significant decrease in absolute leg weight in 5-week-old DON-treated animals is in accordance with the decreased body weight performance in DON-treated animals seen over the whole experimental period. DON therefore seems to decrease weight gain in the form of muscles or bones. Interestingly, the decreasing effect of DON was only shown for broiler legs but not for breast muscle weight, which was not significantly affected by DON treatment in the present study. These findings are in accordance with those of Ghareeb et al. (2016) who observed that absolute and relative weights of the whole carcass and breast were not affected by a diet artificially contaminated with 10 mg DON/kg feed. However, the last-mentioned study did not find statistically significant influences of treatment on leg weights. Likewise, Liu et al. (2011) did not observe statistically significant differences concerning the percentage of breast muscle and thigh muscle in broilers at day 35. However, thigh muscle weights (*n* = 4) were numerically decreased in broilers fed mold-contaminated feed (control 14.8 ± 0.8%, mold 13.9 ± 0.5). The mold-contaminated feed in that study was prepared by replacing half of the non-contaminated maize in the basal diet with mold-contaminated maize containing 450.6 μg/kg aflatoxin B1, 68.4 μg/kg ochratoxin A, and 320.5 μg/kg T-2 toxin (Liu et al. 2011).

The effects of DON on organ weights in chickens in the literature are contradictory. The present study showed that DON treatments significantly decreased both absolute and relative liver weights in week 3 of the experiment. Awad et al. (2006b) found significantly decreased absolute liver weights and numerically decreased relative liver weights in 21-day-old broilers fed a 5 mg/kg DON-contaminated diet. Another study of Awad et al. (2006a) feeding 10 mg DON/kg feed to ROSS broilers until day 42 of life showed that the relative liver weights (*n* = 15) were numerically decreased, compared to those of the control group. However, Dänicke et al. (2002) did not find any difference in liver weight, relative to body weight, in a study feeding *Fusarium* toxin-contaminated maize containing DON and ZON to laying hens (Dänicke et al. 2002). Another study testing five diets, with increasing DON concentrations from 0.0 to 14.0 mg/kg diet with and without the addition of a mycotoxin binder in male broilers from day 1 to day 35 of age, also did not report statistically significant differences in liver weights, although a trend for mycotoxin-contaminated wheat in a quadratic fashion was shown (Dänicke et al. 2003). In broiler breeders, no effect of diet in the relative weight of liver was detected in a study with DON as a major contaminant (12.6 mg/kg feed) (Yegani et al. 2006). Moreover, an experiment including *Fusarium*-infected wheat in the diet of broilers, reaching up to 2.5 mg deoxynivalenol/kg DM, did not report statistically significant effects for liver weights (Dänicke et al. 2007). Another study with three dietary treatments (control, 1 mg DON/kg, 5 mg DON/kg) did not reveal significant effects of DON treatment in absolute and relative liver weights (Awad et al. 2011). In contrast, Yunus et al. (2012) reported that the liver weight in the second week of exposure increased for broilers fed a low DON diet (1.68 mg DON/kg; 0.145 mg zearalenone/kg), than for the basal diet (Yunus et al. 2012).

Altogether, low to moderate doses of DON significantly affect DMI, BW, and carcass traits of broilers. Moreover, DON treatment significantly increased the ratio of WI/DMI. As this can be an indicator for possible nephrotoxicity and thereby the linkage to wet litter problems, further research is warranted to clarify the nephrotoxic potential of DON in broilers.
